# Prevalence and risk factors of syphilis infection among drug addicts

**DOI:** 10.1186/1471-2334-5-33

**Published:** 2005-05-17

**Authors:** Norbert Scherbaum, Bernhard T Baune, Rafael Mikolajczyk, Thomas Kuhlmann, Gerhard Reymann, Martin Reker

**Affiliations:** 1 Department of Psychiatry and Psychotherapy, University Hospital Essen, Germany; 2 Mental Health Epidemiology, Department of Psychiatry and Psychotherapy, University of Muenster, Albert-Schweitzer-Str. 11, 48129 Muenster, Germany; 3 Department of Public Health Medicine; School of Public Health, University of Bielefeld, Germany; 4 Hospital of Psychosomatic Medicine, Bergisch-Gladbach, Germany; 5 Centre for Psychiatry, Psychotherapy, and Psychosomatics, Dortmund, Germany; 6 Centre for Psychiatry and Psychotherapeutic Medicine at Gilead Hospital, Bethel, Bielefeld, Germany

## Abstract

**Background:**

Recent epidemiological data show an increased trend of official estimates for syphilis infection in the general population. Many of the infected cases remain undetected leaving an underestimation of the true prevalence of syphilis in the general population, but also among subpopulations such as illicit drug users. There is limited epidemiological data published on the proportion and risk factors of syphilis infections associated with illicit drug abuse.

**Methods:**

Illicit drug addicts (n = 1223) in inpatients units in Germany were screened (2000–01) for syphilis and interviewed regarding patterns of drug use and sexual behaviour. TPHA-test for initial screening and FTA-ABS-IgM test in TPHA-positive patients were used.

**Results:**

In total, TPHA-tests were positive in 39 (3.3%) and 7 patients (0.6%) were IgM positive. The prevalence rate for syphilis in males was 1.9% and for women it was 8.5%. Female patients were 4.56 (CI 95% 2.37–8.78) times more likely to have a positive TPHA test than males. Sexual behaviours such as high number of sexual partners, sex for drugs/money, sex on the first day were associated with syphilis infection only in women. Females with frequent sex for drugs or money had 4.31 (CI 95% 2.32–8.52) times more likely a reactive TPHA test than remaining patients. Neither the sociodemographic factors nor sexual behaviour were statistically significant associated with syphilis infection among men at all.

**Conclusion:**

Our data suggest the need for screening for syphilis among these illicit drug users in inpatient settings, in particular among sexual active women. This conclusion is corroborated by the finding of increasing numbers of syphilis infections in the general population. The identification of syphilis cases among drug addicts would give treatment options to these individuals and would help to reduce the spread of infection in this population, but also a spread into heterosexual populations related to prostitution.

## Background

World-wide, the incidence and prevalence of syphilis differ due to region, ethnic factors, gender and socio-economic factors [1-5]. In the 90s the yearly incidence of syphilis in Germany decreased to 1.4 cases / 100,000 inhabitants according to official numbers. However, a sentinel surveillance conducted in 1994 showed that 84% of the syphilis cases were not reported and thus, the real incidence could be as much as six times higher [5]. More recently an inverse trend of official estimates was observed with incidence increased to 3.1 cases / 100,000 inhabitants in 2002, but the increase was related only to infections acquired in the sample of men who have sex with men [6].

There are few epidemiological data published on the proportion of syphilis infections associated with illicit drug abuse. According to reports from the USA, drug addicts, especially injecting drug users (IDU), are a high-risk group for syphilis infection [7]. Gourevitch et al. reported 1996 a six year follow-up investigation of 790 opiate addicts in methadone maintenance treatment. In their study, 4.4% of the study subjects had antibodies against syphilis, and the yearly incidence of that infection was 5.7 per 1000 [7]. The prevalence of syphilis in the sample of opiate drug addicts in Germany is not reported so far. Along with abandoned general screening strategies for syphilis at admission to general psychiatric departments [8], screening for syphilis at admission to detoxification wards is no longer clinical routine in most German hospitals, despite severe complications of untreated syphilis [9,10].

## Objectives

The goal of our study was to estimate the prevalence of syphilis in the population of illicit drug addicts, mainly opiate addicts, admitted to detoxification wards in psychiatric hospitals. Furthermore, risk factors associated with syphilis infection were investigated to identify individuals at high risk for syphilis infection in this population.

## Methods

### Study population

Our study was performed at detoxification wards for drug addicts in eight hospitals in the federal state of North Rhine-Westphalia, Germany. Overall, from 1529 eligible patients admitted to any of these detoxification wards during a two years period (2000–2001) 1309 patients participated in the study. Of these patients 86 (7%) individuals left hospital within less than 24 hours or were recurrent admissions and were excluded. Finally, there were 1223 patients in the study, which accounted for an overall response rate of 80%.

All patients were interviewed by physicians using a standardised questionnaire and subsequently tested for syphilis infection. The topics of the questionnaire were: patterns of drug use, sexual risk behaviour and known infection status for Treponema pallidum (syphilis), HIV, Hepatitis B-virus (HBV) and Hepatitis-C-virus (HCV), or Neisseria gonorrhoeae (Gonorrhoea). In addition, sociodemographic data were obtained including country of birth (or ethnic origin) and time of immigration to Germany. The study was approved by the local ethics committee of the University of Essen, Germany, and all patients gave informed consent for participation in the study. Patients identified with active syphilis obtained a standard treatment for syphilis. Penicillin G was applied intravenously due to unknown state and duration of the syphilis infection [11] and potential simultaneous HIV-infection. [12].

### Microbiological testing

German syphilis screening guidelines allow for a two stage testing procedure [11]. Firstly, we used the TPHA as a screening test for Treponema pallidum (Mast TPHA kit, Mast Laboratories, UK). According to producer information the sensitivity of this test is 98.2%, the specificity is 99.7% [13,11]. Secondly, patients with reactive TPHA test were tested using a FTA-ABS IgM-test for confirmation of positive Treponema pallidum. Thus, patients with positive TPHA and FTA-ABS IgM were considered in need of treatment (excluding cases with already initiated treatment before admission). Patients with positive TPHA but negative FTA-ABS IgM were considered either as successfully treated (if there was history of treatment) or as spontaneously recovered from the infection (if there was no history of treatment). VDRL tests were carried out in treated patients to evaluate treatment effects after 3, 6, and 12 months [11]. These follow-up results were not considered in the study.

### Data analysis

Data were entered and analysed with SPSS, Version 11.0 [14]. The prevalence of syphilis infection was determined by calculating the ratio of TPHA-test positives and all tested drug addicts stratified for gender. Risk factors associated with syphilis infection were analyzed by the use of logistic regression analysis adjusted for age and gender.

## Results

### Characteristics of the sample

In total, 1223 illicit drug addicts with a mean age of 30.1 years participated in the study of which 951 were males (77.8%) and 272 females (22.2%). 991 patients (81.6%) were born in Germany.

Nearly all (n = 1186; 97%) of the study population were opiate addicts. The length of opiate addiction in the sample ranged between 1 month and 37 years with the median of 8 years, and there was a relatively equal proportion of users with 1, 2 or 3 (up to 10) years history of use.

425 patients (34.8%) were in a methadone maintenance treatment program at admission with a duration of 2 years (SD 2.1)) on average. 1068 (87.5%) patients reported heroin abuse in the last three months, the majority of them (n = 654) by i.v.-injection. Recent cocaine abuse was reported by 612 patients (55.4%), mostly intravenously (n = 403). A stable partnership over the last year before admission was reported by 265 (21.7%) patients.

### Self-reported infection status and sexual behaviour

A known Hepatitis-C-infection was reported by 65.4% of the subjects, 26.9% knew of a previous Hepatitis-B-infection, 2.3% reported being HIV-positive, 2.4% admitted having had at some time gonorrhoea, and 2.5% reported a former or current syphilis-infection. According to the self-reported sexual behaviour in our sample in table 1, a higher proportion of female (22.7%) than male (4.5%) participants had frequently sex in exchange for drugs or money. Frequent sex on the first day of meeting a partner was also more frequent in females (18.9%) than in males (2.2%). This type of sexual behaviour indicates a higher proportion of prostitutes among females than males (table 1).

**Table 1:** Sero-prevalence of syphilis antibodies (according to TPHA-Test) among inpatient drug addicts by gender, sociodemographic factors and sexual behaviour

	**Male**			**Female**		
	N with test	% with reactive TPHA -Test	P	N with test	% with reactive TPHA -Test	p ^1^

**Overall**	890	1.9		259	8.5	
**Age groups**			0.55			0.05
below 21 years	56	1.8		23	13.0	
21–30 years	406	1.7		128	6.3	
31–40 years	356	1.7		80	6.3	
over 40 years	72	4.2		28	21.4	
**Illicit drug abuse**			0.28			0.13
no or <2 y	91	4.4		33	0	
2 to 4 y	193	1.0		45	6.7	
5–7 y	106	1.9		31	16.1	
>7 y	500	1.8		150	9.3	
**Migration background**			0.18^4^			0.37^4^
Native German	694	1.6		233	9.0	
Migrants	196	3.1		26	3.8	
**Stable partnership**			0.60			0.60
no or <1 year	558	1.8		142	9.9	
1 to 4 years	171	1.2		72	8.3	
≥ 5 years	150	2.7		41	4.9	
**Number of sexual partners in the last 5 years**			0.61			0.00
0–1	247	1.6		49	2.0	
2–5	315	2.2		86	3.5	
6–20	192	1.0		33	0	
>20	136	2.9		91	19.8	
**Sex for drugs/money**			0.52			0.00
never	826	1.8		147	2.7	
seldom	22	0		30	0	
frequent	22	4.5		75	22.7	
**Sex on the first day**			0.89			0.00
never	502	1.6		120	1.7	
seldom	215	1.9		41	2.4	
frequent	137	2.2		90	18.9	
**Condom use with risky partner**			0.53			0.01
never	232	1.3		73	1.4	
up to half of the cases	166	1.2		34	2.9	
in 3/4 of the cases	33	0		14	21.4	
nearly always	182	1.6		73	15.1	
no risky intercourse	236	3.0		52	7.7	

p-value of chi square test or Fisher's exact test, respectively;

### Syphilis-infection status based on testing

TPHA-testing was carried out in 1181 patients of which 39 (3.3%) tests were positive (female n = 22; male n = 17). The distribution of TPHA positive cases ranged from 0% to 8.6 across the 8 participating hospitals. In 24 out of the 39 TPHA-positive cases (61.5%), patients were aware of a former syphilis infection. In 6 cases a former syphilis infection was reported by the patient but the TPHA-test was not reactive. The specific IgM-test for current infection was positive in 7 out of the 39 TPHA-positive cases (0.6% of all tested patients), indicating current syphilis infection (female n = 4; male n = 3). Five out of these 7 patients were not aware of their current infection. Nine out of 32 TPHA positive but FTA-ABS IgM-negative patients did not know about an earlier infection (29%, 3 of 14 men and 6 of 18 women).

### Prevalence of syphilis

The proportion of patients with reactive TPHA is presented in Table 1 showing results stratified for gender. Women had a 4.56 (95% CI 2.37–8.78) times higher risk for a reactive TPHA-test than men. Furthermore, women's sexual behaviour, such as high numbers of sexual partners, sex for drugs/money, and sex on the first day, was associated with syphilis infection. Contrary, none of the figures for men in Table 1 showed any statistically significance. Condom use in men indicated no clear trend for risky or healthy practice, in contrast to women who mostly used condoms at sexual intercourse. The duration of the methadone maintenance program (MMTP) showed no statistically significant difference (p = 0.41) between patients with reactive TPHA (mean duration of MMTP 2.4 y, SD 2.8) and those without reactive TPHA (mean duration of MMTP 2.0 y, SD 2.1).

### Risk factors for syphilis

Table 2 presents a logistic regression analysis for the association between sociodemographic and behavioural parameters and syphilis infection. Apart from gender, no other demographic factor such as age or ethnic background showed any statistically significant associations with syphilis infection. Sexual behaviour was related to infection status as regular sex on the first day meeting a partner and high numbers of sexual partners were statistically significant associated with syphilis infection. Since risk factors differed between women and men, we stratified the results by gender. There were no consistent effects of age or drug abuse history on risk for reactive TPHA apart from an increased risk for older women. Sexual behaviour was associated with an increased risk for syphilis infection in women but not in men; however, even within women the increased risk was limited to women with regular sex for drugs. Females with frequent sex for drugs or money had 4.31 (CI 95% 2.32–8.52) times more likely a reactive TPHA test than remaining patients. Frequent sex on the first day of meeting a partner was statistically significant associated with syphilis infection in females (OR 17.8; 95%CI, 3.8–83.38), but not in males (OR 1.43; 95%CI, 0.37–5.5). There was an increased risk in women who used condoms more consistently, but after adjustment for regular sex for drugs this effect was not significant any more (not shown). No gender differences were found for the association of syphilis infection and migration or the existence of a stable partnership, respectively.

**Table 2:** Association of sociodemographic factors, clinical parameters and sexual behaviour with syphilis infection among 1223 drug addicts

	**Syphilis infection (positive for TPHA)**		
	OR*	**95%CI**	**p-value**
**Gender**			
Male	1	[reference group]	<0.00
Female	4.56	[2.37–8.78]	
**Age groups**			
below 21 y	1	[reference group]	
21–30 y	0.59	[0.19–1.85]	0.363
31–40 y	0.59	[0.18–1.94]	0.383
over 40 y	1.94	[0.56–6.71]	0.295
**Illicit drug abuse**			
no or <2 y	1	[reference group]	
2 to 4 y	0.89	[0.23–3.53]	0.872
5–7 y	1.88	[0.52–6.79]	0.334
>7 y	0.96	[0.32–2.92]	0.945
**Migration background**			
native German	1	[reference group]	
Migrants	1.51	[0.63–3.62]	0.36
**Stable partnership**			
> 5 years	1	[reference group]	
1 to 5 years	1.17	[0.46–2.97]	0.94
<1 year	1.04	[0.35–3.16]	0.74
**Number of sexual partners in the last 5 years**			
0–1	1	[reference group]	
2–5	1.71	[0.57–5.17]	0.34
6–20	0.69	[0.13–3.67]	0.67
> 20	5.85	[2.07–16.5]	0.00
**Sex for drugs/money**			
never	1	[reference group]	
frequent	8.81	[3.66–21.19]	0.00
**Sex on the first day of meeting a partner**			
never	1	[reference group]	
seldom	1.31	[0.44–3.9]	0.63
frequent	4.98	[2.22–11.19]	0.00

OR denotes Odds ratio; CI denotes confidence interval; *adjusted for sex and age (where appropriate)

## Discussion

The sero-prevalence of syphilis (according to TPHA) of 3.3% in our study is comparable to the findings of studies carried out in the USA among drug abusers in treatment facilities [7,15,16], but it is much lower than 23% among injecting drug users in Bangladesh [17]. However, only 1 in 5 of the TPHA positive patients in our study (0.6% of all screened patients) was in need of treatment, all other patients were already treated or recovered from the disease; these proportions were not reported for the other countries and may significantly differ.

According to our results, women were more often affected than men; the risk was especially increased in women having commercial sex. A study among IDUs in Spain showed a 4 times higher prevalence of syphilis and other STDs among women than men [16]. Similar to our results, syphilis was associated in this study with commercial sex in women.

We could not identify any significant risk factors for syphilis in male IDUs in our sample. We neither found a higher risk in immigrants nor in HIV positive patients. An increased risk for syphilis among immigrants especially from East European countries has been expected based on the reports of a higher prevalence of syphilis in these countries [18]. Although in our study about one third of immigrants (75 out of 205) came originally from the former USSR, no increased risk for syphilis was detected for this group. In our study intravenous drug use (heroin or cocaine) was not a proven risk factor for previous or current syphilis infection, whereas it was observed in the study by Rolfs et al. [19].

Contrary to our results an Italian study on heroin addicts with a 6-year follow-up period did not find any cases of syphilis infection [20]. The possible explanation for this difference is the higher proportion of patients with past or current commercial sex activity in our study. Additionally, the differences in prevalence can be caused by the different recruitment procedures used in both studies. While we performed a cross-sectional study, the Italian group used a cohort design. The follow-up of drug addicts over years usually requires patients in long-term contact with the support system. Such patients in follow-up or treatment settings might have a lower risk for the syphilis infection. However, this hypothesis could be not corroborated by our data, because we could not demonstrate a protective effect of the participation in substitution programs on the syphilis sero-status. An additional aspect is related especially to the infectious nature of syphilis. The Italian data were based on an investigation in one city only. In our study the prevalence of syphilis (TPHA positive tests) ranged between 0% and 8.9% for the eight study centres. This shows that syphilis may not spread efficiently between clusters of drug addicts in different cities and it may account for the differences between the Italian data and our study.

Given the complexity of long term complications, our estimate of prevalence does not provide sufficient information for a cost-effectiveness analysis. An analysis by Banger et al. supporting to abandon the general screening strategy at admission to psychiatric hospitals was based on 0.07% screening tests being positive [8]. Our data show higher numbers of screening positives in this high risk group setting compared to the study results of Banger [8]. Thus, syphilis screening for this high risk group should be favoured.

There are some important limitations to our study. Because of the very low prevalence of positive FTA-ABS-IgM tests confirming active syphilis, we had to use TPHA positive cases for our analysis of risk factors, i.e. including all patients with a past and current syphilis infection in the same group. This requires the assumption that the characteristics or behaviour of the patients with a past infection still do reflect their risk status. With respect to gender and women's sexual risk behaviour, the assumption was corroborated by the agreement between risk prediction based on TPHA and FTA-ABS-IgM. Nevertheless, the failure to determine any further risk factors can be caused by the misrepresentation of the risk factors in the analysis based largely on patients who had syphilis in the past. Some cases might have remained undetected (false negative) but it is unlikely that this would be associated with any particular risk factors. On the other side the false positive cases would have the effect of diminishing estimate of the true increased risk.

## Conclusion

In conclusion our data underline the importance to screen drug addicts in inpatient treatment settings for syphilis as these patients represent a high risk group for sexually transmitted diseases. In particular sexual active women having high numbers of sexual partners were at highest risk for syphilis infection. Especially infected prostitutes may act as a multiplier in the dynamics of spread of infection into the heterosexual population.

The decision to abandon a general screening for syphilis in many hospitals was to our knowledge neither based on a formal cost-effectiveness analysis nor on the analysis of the impact on the incidence of syphilis in a society. Taking into account increasing numbers of syphilis cases worldwide, we would suggest at least a screening for syphilis among high risk opiate drug addicts in hospitals where general screening for syphilis was abandoned. Furthermore, it might useful to suggest testing for gonorrhea and chlamydia as well as for syphilis for people in drug treatment programs as a significant amount of gonorrhea and chlamydia is asymptomatic, especially in women.

Although we studied only the situation in Germany, similar conditions may apply to other developed countries.

## Declaration of competing interests

The author(s) declare that they have no competing interests.

## Authors' contributions

NSCH, BTB and RM contributed to the study design, data collection and analysis, and preparation of the draft manuscript; TK, GR, and MR were responsible for conceptualisation of the study and for coordination of data collection; all authors took part in revising the manuscript.

## Pre-publication history

The pre-publication history for this paper can be accessed here:



## References

[B1] Aseffa A, Ishak A, Stevens R, Fergussen E, Giles M, Yohannes G, Kidan KG (1998). Prevalence of HIV, syphilis and genital chlamydial infection among women in north-west Ethiopia. Epidemiology & Infection.

[B2] Dada AJ, Ajayi AO, Diamondstone L, Quinn TC, Blattner WA, Biggar RJ (1998). A serosurvey of Haemophilus ducreyi, syphilis and herpes simplex virus type 2 and their association with human immundeficiency virus among female sex workers in Lagos, Nigeria. Sexually Transmitted Diseases.

[B3] Kambarami RA, Manyame B, Macq J (1998). Syphilis in Murewa district, Simbabwe: an old problem that rages on. Central African Journal of Medicine.

[B4] Prange HW, Ritter G (1981). [The epidemiology of neurosyphilis]. Nervenarzt.

[B5] Epidemiological Bulletin (1997). [Current data and information on infectious diseases: current implications]. Robert Koch Institute.

[B6] Epidemiological Bulletin (2003). [Current data and information on infectious diseases and Public Health: Syphilis in Germany in 2002]. Robert Koch Institute.

[B7] Gourevitch MN, Hartel D, Schoenbaum EE, Selwyn PA, Davenny K, Griedland GH, Klein RS (1996). A prospective study of syphilis and HIV infection among injection drug users receiving methadone in the Bronx. American Journal of Public Health.

[B8] Banger M, Olbrich HM, Fuchs S, Gastpar M (1995). [Cost-effectiveness of syphilis screening in a clinical for general psychiatry]. Nervenarzt.

[B9] Luger A, Gschnait F, Niebauer G (1987). Economical aspects of routine screening for syphilis in Viennese municipal hospitals. Wiener Klinische Wochenschrift.

[B10] Gjestland T (1955). The Oslo study of untreated syphilis; an epidemiologic investigation of the natural course of the syphilitic infection based upon a re-study of the Boeck-Bruusgaard material. Acta Derm Venereol.

[B11] Schneede P, Hofstetter A Infectious disease working group of the German Society of Urology: Guidelines for the diagnostic and treatment of STDs. http://leitlinien.net.

[B12] French P, Syphilis Guidelines Revision Group. UK national guidelines on the management of late syphilis 2002. http://www.mssvd.org.uk.

[B13] Egglestone St, Ashley I (1995). Sensitivity and specificity of TPHA test. Supra Regional Treponemal Serology Lab, PHL, Bristol.

[B14] SPSS for Windows, Release 11.01. SPSS Inc Headquarters, 233 S Wacker Drive, 11th floor Chicago, Illinois 60606.

[B15] Hwang LY, Ross MW, Zack C, Bull L, Rickman K, Holleman M (2000). Prevalence of sexually transmitted infections and associated risk factors among populations of drug abusers. Clin Infect Dis.

[B16] Muga R, Roca J, Tor J, Pigem C, Rodriguez R, Egea JM, Vlahov D, Munoz A (1997). Syphilis in injecting drug users: clues for high-risk sexual behaviour in female IDUs. Int J Std AIDS.

[B17] Azim T, Bogaerts J, Yirell DL, Banerjea AC, Sarker MS, Ahmed G, Amin MM, Rahman AS, Hussain AM (2002). Injecting drug users in Bangladesh: prevalence of syphilis, hepatitis, HIV and HIV subtypes. AIDS.

[B18] Atlani L, Carael M, Brunet JB, Frasca T, Chaika N (2000). Social change and HIV in the former USSR: the making of a new epidemic. Soc Sci Med Jun.

[B19] Rolfs RT, Goldberg M, Sharrar RG (1990). Risk factors for syphilis: cocaine use and prostitution. American Journal of Public Health.

[B20] Lugobini F, Quaglio G, Mezzelani P, Lechi A (2002). No positive tests for syphilis in 6 years of observation among heroin drug users in north-eastern Italy. Addiction.

